# Hydrogen-bonds structure in poly(2-hydroxyethyl methacrylate) studied by temperature-dependent infrared spectroscopy

**DOI:** 10.3389/fchem.2014.00010

**Published:** 2014-03-12

**Authors:** Shigeaki Morita

**Affiliations:** Department of Engineering Science, Osaka Electro-Communication UniversityNeyagawa, Japan

**Keywords:** PHEMA, hydrogen-bond, glass transition, infrared spectroscopy

## Abstract

Hydrogen-bonds structure in poly(2-hydroxyethyl methacrylate) (PHEMA) were investigated by means of temperature-dependent infrared (IR) spectroscopy. Spectral variations involved with the OH…OH and C=O…HO types of hydrogen-bonds were found around the glass transition temperature of 80°C. Hydrogen-bonds among the hydroxyl groups gradually dissociate with increasing temperature. In contrast, discontinuous variation in the carbonyl bands was observed around the glass transition temperature. An association of the C=O…HO type of hydrogen-bond with increasing temperature above the glass transition temperature was revealed. These were concluded from the present study that hydrogen-bonds among the hydroxyl groups in each side chain terminal suppress the main chain mobility in the polymer matrix below the glass transition temperature, while the dissociation of the OH…OH type of hydrogen-bonds induces the association of the C=O…HO type of hydrogen-bond. As a result, the mobility of the main chain is induced by the change in hydrogen-bonds structure at the glass transition temperature.

## Introduction

Poly(2-hydroxyethyl methacrylate) (PHEMA) contains one carbonyl (C=O) and one hydroxyl (OH) groups on each side chain (Montheard et al., 1992). The OH group acts as both proton donor and proton acceptor, while the C=O group as only proton acceptor (Jeffrey, 1997; Marechal, 2007). Thus, both OH…OH and C=O…HO types of hydrogen-bonds are acceptable in PHEMA. Not only dimer structure (OH…OH) but also aggregates structure (…OH…OH…OH…) have been found in many systems including liquid alcohols (Kristiansson, 1999; Ohno et al., 2008) and solid polymers (Morita et al., 2008, 2009). Such the hydrogen-bonds structure in polymers plays important roles for their macromolecular functions in artificial polymers (Brunsveld et al., 2001) as well as biopolymers (Watanabe et al., 2006, 2007). Our recent study revealed that 47.3% of the OH group on the PHEMA side chain terminal are engaged in the OH…O=C type of hydrogen-bond, while the remaining 53.7% contributes to the OH…OH type of hydrogen-bond at ambient temperature (Morita et al., 2009).

In the present study, change in hydrogen-bonds structure in PHEMA in the vicinity of glass transition temperature was explored by means of temperature-dependent infrared (IR) spectroscopy (Perova et al., 1997; Morita et al., 2008; Morita and Kitagawa, 2010). Although PHEMA is water insoluble, large amounts of water is sorbed into a PHEMA matrix with an equilibrium water content of ca. 40 wt% (Tanaka et al., 2002). A dry PHEMA solid is brittle, since its glass transition temperature is higher than ambient temperature. On the other hand, a PHEMA hydrogel, i.e., a water sorbed PHEMA, becomes soft material, because its glass transition temperature is reduced to be lower than ambient temperature (Roorda et al., 1988). These imply macromolecular properties in PHEMA are characterized by non-covalent interactions of hydrogen-bonds among the polymer chains as well as the hydrated water molecules.

## Materials and methods

An atactic PHEMA with a viscosity-averaged molecular weight of ca. 3.0 × 10^5^ was purchased from Aldrich and used without further purification. A glass transition temperature of the PHEMA sample evaluated by differential scanning calorimetry (DSC) was 80°C, which was performed using a PerkinElmer Pyris 6 at a heating rate of 10°C min^−1^. An evidence of crystalline phase in the solid was not detected by DSC, demonstrating that the PHEMA sample used in the present study is amorphous. A film sample was prepared on a calcium fluoride substrate by solvent casting from a methanol solution. A thickness of the film sample was controlled as all the IR signals in an absorbance unit become less than 1. The film sample was enough dried at an ambient temperature before the measurement. Temperature-dependent IR spectra of the PHEMA film were collected over a temperature range of 25–150°C with an increment of 1°C using a Fourier transform IR spectrometer (Varian, FTS-3000) equipped with a deuterated triglycine sulfate detector at a nitrogen atmosphere. A total of 128 scans were co-added to obtain each spectrum.

## Results

**Figure 1:**
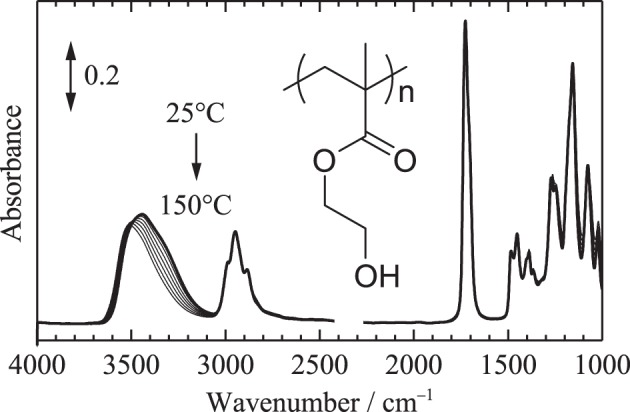
**Temperature-dependent IR spectra of PHEMA in the range of 25–150°C measured with an increment of 1°C (all the spectra are not shown here)**. Bold line corresponds to the spectrum at 25°C.

Figure 1 shows temperature-dependent IR spectra of the PHEMA film. No IR signals arising from the residual solvent of methanol and hydrated water from ambient air were detected, representing that a sufficiently dry film was obtained at the nitrogen atmosphere. This represents O-H stretching band in the spectra is arising from only PHEMA. Assignments of IR absorption bands in the spectrum of PHEMA have been reported previously (Ferreira et al., 2000; Morita et al., 2009). The assignments are summarized in Table 1. A large spectral shape variation in the O-H stretching region around 3700–3100 cm^−1^ was observed, while only weak variations were detected in the other spectral regions. Detailed spectral variations in the O-H stretching and the C=O stretching regions are discussed below.

**Table 1:** **Assignments of selected IR absorption bands in PHEMA**.

**Wavenumber/cm^−1^**	**Assignments**
3666	Free OH (trans)
3624	Free OH (gauche)
3534	Hydrogen-bonded OH with C=O (OH…O=C), dimer OH (OH…OH)
3434	First overtone of C=O stretching
3320	Aggregates OH (… OH…OH…OH …)
1730	Free C=O
1703	Hydrogen-bonded C=O with OH (C=O…HO)

### O-H stretching region

**Figure 2:**
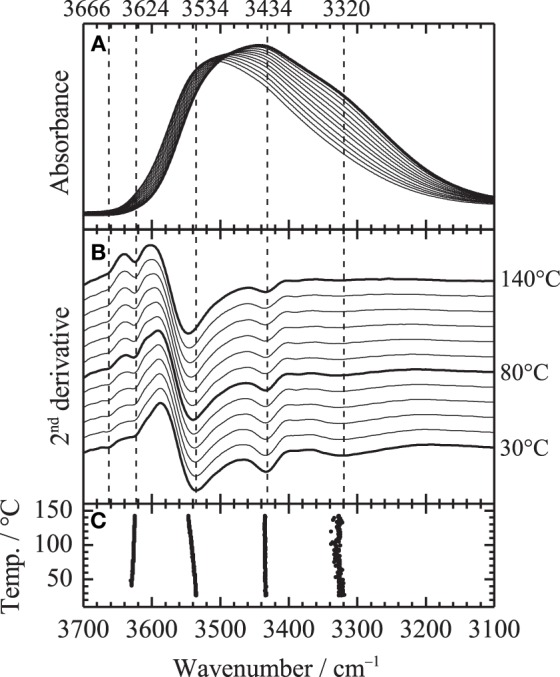
**(A)** Close up of Figure 1 in the O-H stretching region, **(B)** second derivative spectra and **(C)** peak position of the second derivative spectra. Bold line in **(A)** corresponds to the spectrum at 25°C.

Figure 2A shows the temperature-dependent IR spectra of PHEMA in the O-H stretching region (close up of Figure 1). Second derivative spectra calculated from the obtained spectra shown in Figure 2A and peak positions of the second derivative spectra plotted as a function of temperature are also depicted in Figures 2B,C, respectively. At least five contributions around 3666, 3624, 3534, 3434, and 3320 cm^−1^ are identified in the O-H stretching region. Our recent study using model compounds of methanol, methyl acetate and 2-hydroxyethyl methacrylate monomer revealed their assignments as summarized in Table 1 (Morita et al., 2009). Although very weak signals in the obtained spectra, the bands arising from free OH, i.e., OH group not donating hydrogen-bond, are clearly identified at 3666 and 3624 cm^−1^ in the second derivative spectra above the glass transition temperature of 80°C.

**Figure 3:**
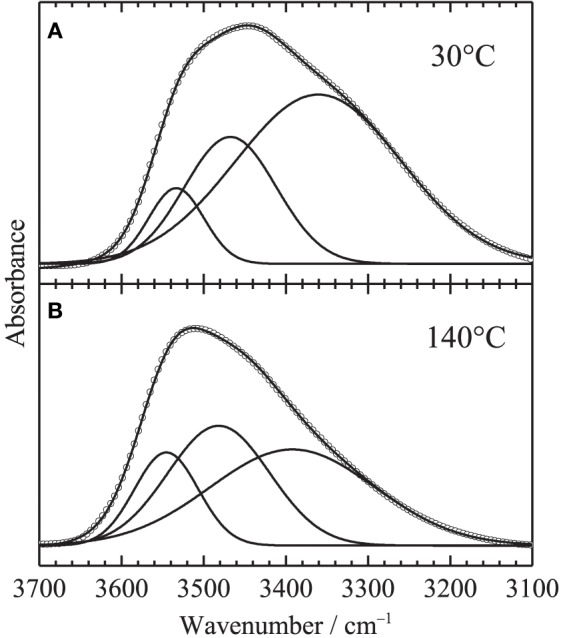
**Typical results of the curve fitting in the O-H stretching region assuming three Gaussian components at (A) 30°C and (B) 140°C**. Circles and lines in the figure correspond to observed data and fitted lies with the three components.

**Figure 4:**
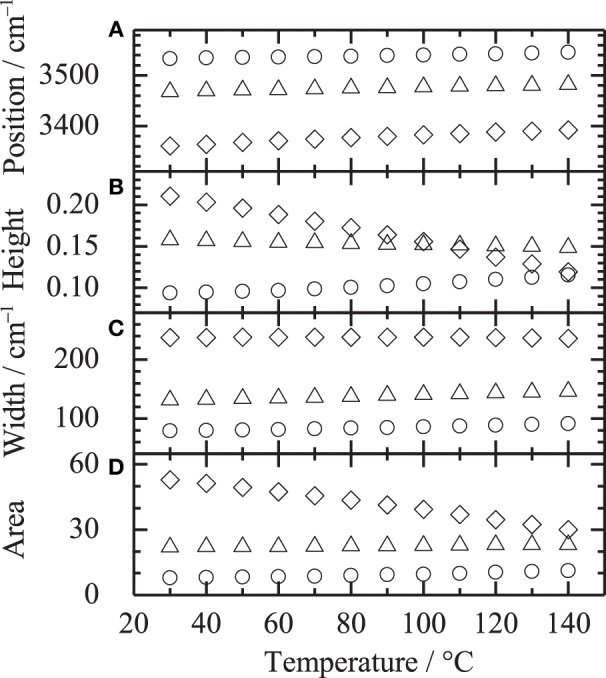
**Fitting parameters of (A) peak position, (B) peak height, (C) peak width and (D) area intensity in the O-H stretching region plotted as a function of temperature**. Symbols of circle, triangle and diamond correspond to the bands around 3534, 3434, 3320 cm^−1^, respectively.

In order to clarify the spectral variations in the O-H stretching region, the spectral shapes in the region were fitted using the following Gaussian functions:

$$A(\nu )=\sum_{i}h_{i}\exp\left(-\text{4ln2}\frac{{\left(\nu -\nu_{i}\right)}^{2}}{w_{i}^{2}}\right)$$

were, *h*_*i*_, ν_*i*_ and *w*_*i*_ are peak height, peak position and peak width, respectively. Figure 3 shows the fitting results at **(A)** 30 and **(B)** 140°C, respectively. All the spectra were well fitted assuming three Gaussian contributions, since the bands at 3666 and 3624 are very weak. Figure 4 depicts the fitting parameters for the O-H stretching region plotted as a function of temperature. Variations of the peak position and the peak width are relatively small, while those of peak height or area intensity, which is calculated as

$$A_{i}=\int A_{i}\left(\nu \right)\text{d}\nu$$

are intense. The intensity variation of the band at 3434 cm^−1^, which is assigned to the first overtone of the C=O stretching, is not clearly identified. In contrast, the intensity of the band at 3320 cm^−1^ gradually decreases with increasing temperature, while that at 3534 cm^−1^ gradually increases. It should be noted that the intensity variations at 3320 and 3534 cm^−1^ are not discontinuous with temperature at the glass transition temperature of 80°C.

### C==O stretching region

**Figure 5:**
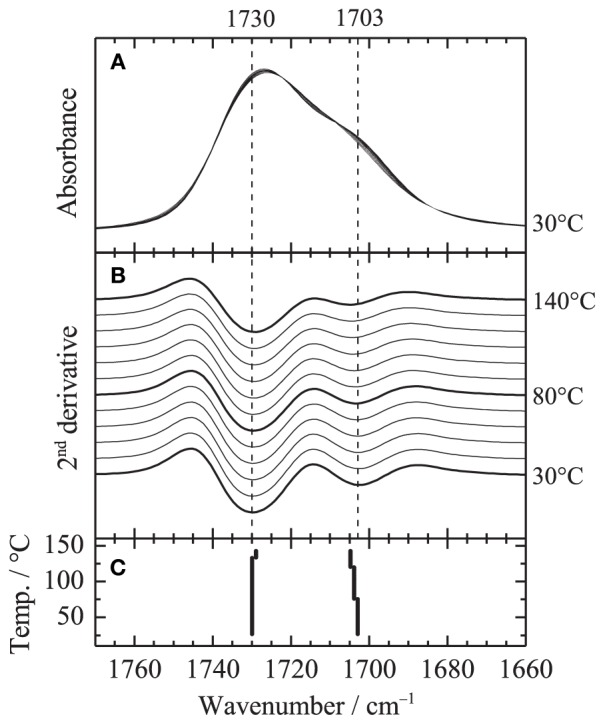
**(A)** Close up of Figure 1 in the C=O stretching region, **(B)** second derivative spectra and **(C)** peak position of the second derivative spectra.

**Figure 6:**
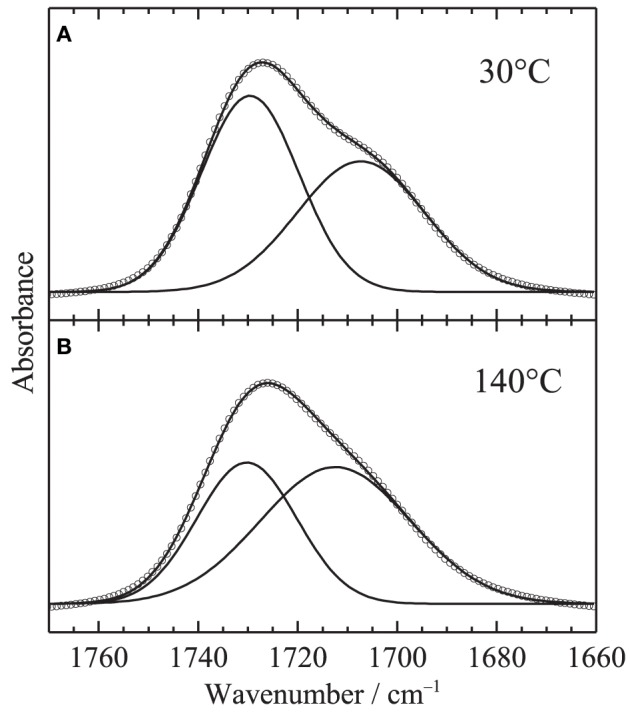
**Typical results of the curve fitting in the C=O stretching region assuming two Gaussian components at (A) 30°C and (B) 140°C**. Circles and lines in the figure correspond to observed data and fitted lies with the two components.

**Figure 7:**
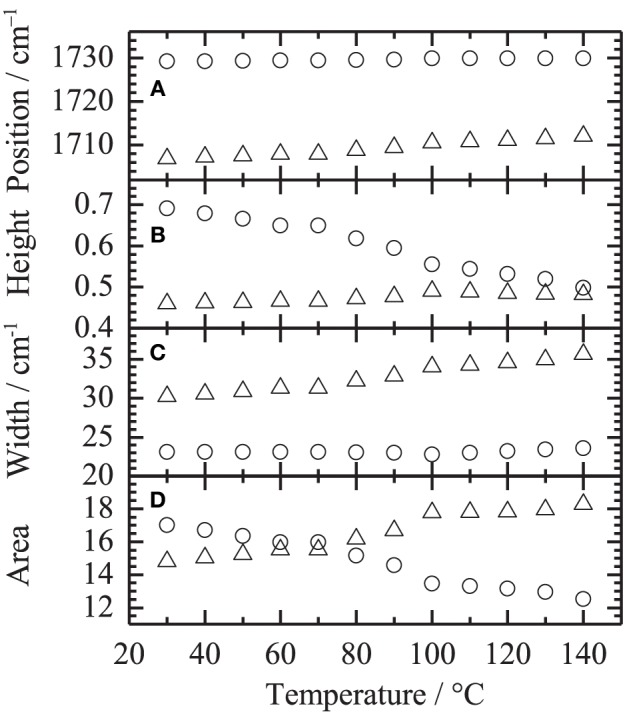
**Fitting parameters of (A) peak position, (B) peak height, (C) peak width and (D) area intensity in the C=O stretching region plotted as a function of temperature**. Symbols of circle and triangle correspond to the bands around 1730 and 1703 cm^−1^, respectively.

Figure 5A shows the temperature-dependent IR spectra of PHEMA in the C=O stretching region (close up of Figure 1). Second derivative spectra and peak positions of the second derivative spectra are also plotted in Figures 5B,C, respectively. Two contributions around 1730 and 1703 cm^−1^ are identified in the C=O stretching region. Assignments of the two bands given in our previous study (Morita et al., 2009) are summarized in Table 1. In order to clarify the spectral variations, the shapes in the region were fitted by two Gaussian components. Figure 6 shows the fitting results at **(A)** 30 and **(B)** 140°C, respectively. All the spectra were well fitted assuming two Gaussian contributions. Figure 7 depicts the fitting parameters for the C=O stretching region plotted as function of temperature. The peak position of the band around 1730 scarcely changed with temperature, whereas that around 1703 cm^−1^ shifts toward to the higher wavenumber as similar to the peak position evaluated by the second derivatives as shown in Figure 5C. The peak width of the band at 1730 also scarcely changed, while that at 1703 cm^−1^ becomes broad with increasing temperature. It is of particular to note that the peak height or the area intensity of the band at 1703 cm^−1^ assigned to hydrogen-bonded C=O discontinuously increases above the glass transition temperature of 80°C, whereas that at 1730 cm^−1^ assigned to free C=O discontinuously decreases at the temperature.

## Discussion

An evidence of gradual dissociation of the OH…OH type of hydrogen-bonds with increasing temperature was found in the O-H stretching region. In contrast, it was found in the C=O stretching region that association of the C=O…HO type of hydrogen-bond occurs discontinuously above the glass transition temperature. The second derivative spectra in the O-H stretching region revealed that the free OH appears above the glass transition temperature.

**Figure 8:**
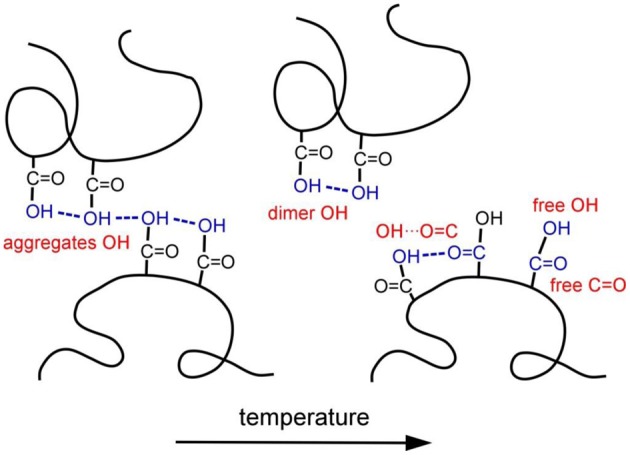
**Schematic illustration of the change in hydrogen-bonds structure in PHEMA induced by temperature**.

A schematic illustration of the change in hydrogen-bonds structure in PHEMA induced by temperature speculated from the spectral variations is described in Figure 8. At ambient temperature, 53.7% of the OH groups in the side chain terminal are associated with each other via the…OH… OH…OH…type of hydrogen-bonds. As a result, mobility of the main chain is expected to be suppressed by the non-covalent interactions among the side chains. However, the aggregates OH are easily dissociated by increasing temperature. At the glass transition temperature, which relates to the mobility of polymer main chain, the OH groups which dissociated with the other OH groups are associated with the C=O groups with the OH…O=C type of hydrogen-bond. It is likely that the mobility of the main chain is induced by the change in the hydrogen-bonds structure at the glass transition temperature.

Glass transition temperature for analogous poly(acrylate)s are generally lower than that for PHEMA as summarized in Table 2 (Morita et al., 2004). Only glass transition temperature for PMMA is higher than that for PHEMA because of non-bulky side chain, which enhances the main chain interaction. Glass transition temperature for PHEMA hydrogels is reduced by increasing a content of water in the matrix (Roorda et al., 1988). These also support the conclusion that the mobility of the PHEMA main chain is induced by the dissociation of the OH…OH type of hydrogen-bonds among the side chains, since water molecules also hydrated to both OH and C=O groups in the PHEMA side chain via the OH…OH and C=O…HO types of hydrogen-bonds (Tsuruta, 2010). Miwa et al. found the evidences of strong OH…OH and C=O…HO types of hydrogen-bonds between the PHEMA side chain and water molecule using NMR spectroscopy (Miwa et al., 2010). In the case of PHEMA hydrogels, it is likely that the hydrogen-bonds among the PHEMA side chains are partially inhibited by water molecules. As a result, glass transition temperature for PHEMA is reduced by the content of water.

**Table 2:** **Typical glass transition temperature for poly(acrylate)s (Morita et al., 2004)**.

	**Side chain structure**	**Typical glass transition temperature/°C**
Poly(2-methoxyethyl acrylate) (PMEA)	-COO(CH_2_)_2_OCH_3_	−50
Poly(ethyl acrylate) (PEA)	-COOCH_2_CH_3_	−24
Poly(*n*-butyl methacrylate) (PBMA)	-CH_3_, -COO(CH_2_)_3_CH_3_	20
Poly(2-hydroxyethyl methacrylate) (PHEMA)	-COO(CH_2_)_2_OH	80
Poly(methyl methacrylate) (PMMA)	-CH_3_, -COOCH_3_	105

### Conflict of interest statement

The author declares that the research was conducted in the absence of any commercial or financial relationships that could be construed as a potential conflict of interest.
